# Symptomatic Long Pauses and Bradycardia due to Massive Multinodular Goiter

**DOI:** 10.1155/2017/4201942

**Published:** 2017-08-06

**Authors:** Amrish Deshmukh, Cevher Ozcan

**Affiliations:** ^1^Department of Medicine, University of Chicago, Chicago, IL, USA; ^2^Section of Cardiology, University of Chicago, Chicago, IL, USA

## Abstract

Sinus node dysfunction with symptomatic bradycardia or chronotropic incompetence is generally an indication for pacemaker implantation. However, in patients with symptomatic sinus bradycardia, the identification and treatment of underlying pathologies may avoid the need for permanent pacemaker implantation. We present a case of carotid sinus syndrome and severe obstructive sleep apnea due to a massive multinodular goiter in a patient who presented with recurrent sinus pauses and syncope. The patient was managed without pacemaker implantation but instead with thyroidectomy resulting in decompression of the carotid sinus and airway and resolution of bradycardic episodes.

## 1. Introduction

Permanent pacemaker implantation is generally indicated in patients with symptomatic sinus node dysfunction. Such sinus node dysfunction may be the result of age related degeneration or secondary to ischemic, infiltrative, endocrinologic, or autonomic diseases [1]. Mass lesions of the head and neck, including multinodular goiters, have been reported to cause bradycardia, pauses, or syncope [2, 3]. Carotid sinus syndrome or autonomic dysfunction may occur when mass effect involves the carotid sinus baroreceptor, airway, or recurrent laryngeal nerve [2]. With surgical decompression or denervation, the cardioinhibitory and vasodepressor response to compression can be reversed [2, 3]. We present the case of a 55-year-old woman with recurrent sinus pauses and syncope who was found to have carotid sinus and airway compression from a massive multinodular goiter.

## 2. Case Presentation

**Figure 1:**
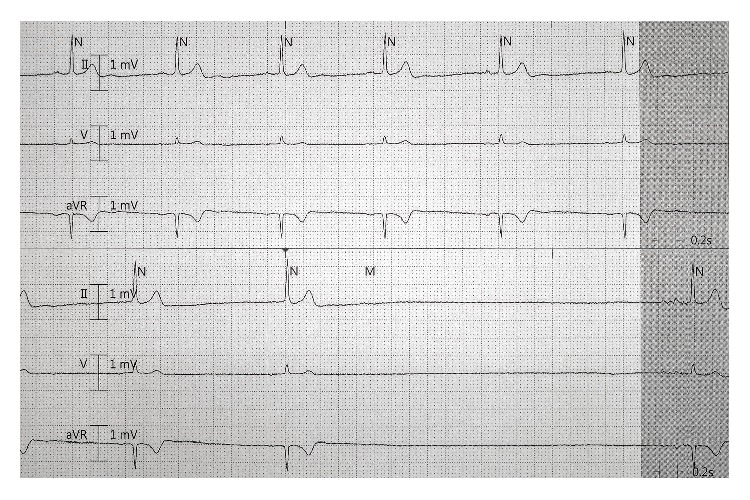
Telemetry rhythm strip demonstrates sinus bradycardia preceding a sinus pause.

A 55-year-old woman with history of bipolar disorder, obstructive sleep apnea (OSA), and multinodular goiter presented with recurrent syncope. The patient also endorsed nocturnal palpitations and several syncopal events associated with voiding. There was no previous history of arrhythmia, conduction disease, or known cardiac structural or functional abnormality. Electrocardiogram demonstrated sinus rhythm with normal atrioventricular nodal and ventricular conduction intervals including PR, QRS, and QTc at baseline. Her sinus rate was 70 to 80 beats per minute at rest. Laboratory analyses including electrolytes and thyroid function were unremarkable. However, inpatient telemetry monitoring demonstrated frequent episodes of sinus bradycardia which were asymptomatic and intermittent long sinus pauses of up to 9 seconds (Figure 1()). These bradycardic events were most frequent and prolonged during sleep and when the patient was noncompliant with continuous positive airway pressure ventilation. Few episodes of sinus tachycardia were also recorded; however no other arrhythmias were present. She was not on any medication to suppress the sinus or atrioventricular nodes. Transthoracic echocardiogram demonstrated normal cardiac structure and function.

**Figure 2:**
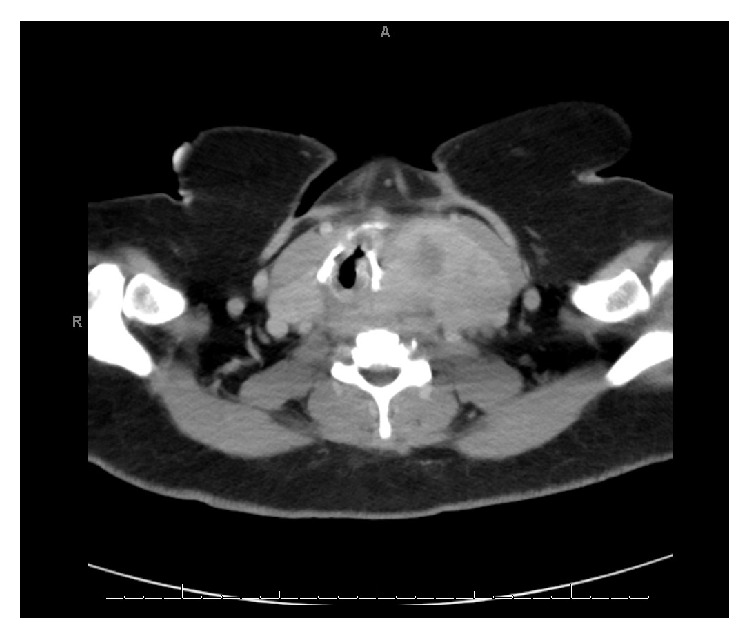
An axial image of the goiter with computed tomography.

**Figure 3:**
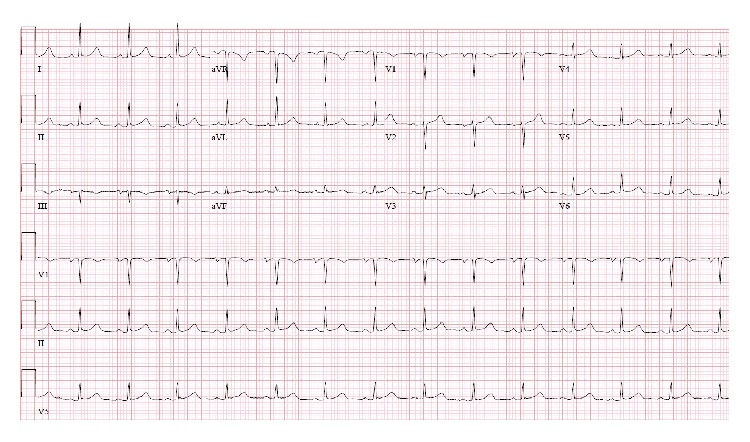
Improved heart rhythm after thyroidectomy.

Computed tomography imaging revealed a massive goiter extending from the nasopharynx to aortic arch causing rightward tracheal deviation (9.1 × 6.1 × 10 cm in greatest dimensions, Figure 2()). Given the potential reversibility of the inciting cause of bradycardia, permanent pacemaker placement was deferred. The patient underwent total thyroidectomy without intraoperative arrhythmia and had complete resolution of sinus bradycardia, sinus pauses, and symptoms. Tissue pathology of the thyroid gland was consistent with benign multinodular goiter. There was no infiltrative disease or malignant tissue invasion to carotid artery, except thyroid gland mass effect or external pressure to local neck structures including carotid. The patient remained in sinus rhythm at heart rate of 80–90 beats per minute during postoperative care. Cardiac monitoring showed no further brady- or tachyarrhythmia during follow-up (Figure 3()).

## 3. Discussion

Carotid sinus compression by malignant masses has been previously described in the literature to produce both cardioinhibitory and vasodepressor responses [2–4]. Carotid sinus compression by a neck mass or the invasion of the tumor in patients with head and neck cancer can cause episodes of bradycardia and intermittent pauses. Carotid body tumors, local tumors such as thyroid cancers, cervical lymphadenopathy, postradiation fibrosis, and internal carotid artery aneurysms, are known to cause carotid sinus sensitivity [5–7]. Although the pathophysiology of this process is well-characterized, it is suggested to be associated with a permanent depolarization of neuronal axons with an increased sensitivity to impulses. However, it is extremely rare for multinodular goiter to cause carotid baroreceptor hypersensitivity leading to symptomatic sinus pauses and bradycardia. Here we present a case of symptomatic sinus pauses and bradycardia due to carotid baroreceptor compression by massive multinodular goiter.

While ventricular demand pacing may be effective in managing cardioinhibitory responses, vasodepressor responses to carotid baroreceptor compression may limit pacemaker utility [3, 8]. In addition, significant vasodepressor symptoms may manifest only after ventricular pacing is instituted [3, 8]. In such patients, sequential atrioventricular pacing appears to alleviate vasodepressor symptoms thought to be caused by atrial contraction against a closed atrioventricular valve [8, 9].

In this patient, the effect of carotid baroreceptor hypersensitivity was compounded by concomitant OSA with pronounced nocturnal bradycardia. The incidence of OSA is increased in patients with massive goiters and OSA is associated with a high burden of nocturnal bradycardia [10, 11]. This bradycardia is thought to be vagally mediated as electrophysiologic studies of patients with OSA are normal [11, 12]. Finally thyroidectomy appears to improve OSA in patients with massive goiter and improvement of OSA with CPAP attenuates nocturnal bradycardia [10, 11]. Therefore, it is plausible that the improvement of OSA by thyroidectomy in this patient helped alleviate her nocturnal bradycardia and sinus pauses. Notably, consistent with the majority of benign goiters, the patient was euthyroid [13].

Our case also illustrates the importance of an investigation for reversible causes in the work-up of symptomatic sinus bradycardia and pauses. Mass lesions of the neck involving the carotid sinus or airway are a potentially modifiable cause of sinus bradycardia and sleep apnea. Removal of the mass may obviate the need for pacemaker placement. Awareness of those triggers and appropriate treatment of underlying causes are optimal therapeutic approaches for the patient with secondary sinus bradycardia and pauses.

## References

[1] Epstein A. E., Di Marco J. P., Ellenbogen K. A. (2008). ACC/AHA/HRS 2008 guidelines for device-based therapy of cardiac rhythm abnormalities: a report of the american college of cardiology/american heart association task force on practice guidelines (writing committee to revise the ACC/AHA/NASPE 2002 guideline update for implantation of cardiac pacemakers and antiarrhythmia devices) developed in collaboration with the american association for thoracic surgery and society of thoracic surgeons. *Journal of the American College of Cardiology*.

[2] Cicogna R., Bonomi F. G., Curnis A. (1993). Parapharyngeal space lesions syncope-syndrome a newly proposed reflexogenic cardiovascular syndrome. *European Heart Journal*.

[3] Patel A. K., Yap V. U., Fields J., Thomsen J. H. (1979). Carotid sinus syncope induced by malignant tumors in the neck: emergence of vasodepressor manifestations following pacemaker therapy. *Archives of Internal Medicine*.

[4] Rothstein S. G., Jacobs J. B., Reede D. L. (1987). Carotid sinus hypersensitivity secondary to parapharyngeal space carcinoma. *Head & Neck Surgery*.

[5] Gunnar Wallin B., Westerberg C.-E., Sundlöf G. (1984). Syncope induced by glossopharyngeal neuralgia: sympathetic outflow to muscle. *Neurology*.

[6] Mehta N., Abdelmessih M., Smith L., Marieb M. (2014). Carotid sinus syndrome as a manifestation of head and neck cancer - case report and literature review. *International Journal of Clinical Cardiology*.

[7] Papay F. A., Roberts J. K., Levine H. L., Wegryn T. L., Gordon T. (1989). Evaluation of syncope from head and neck cancer. *Laryngoscope*.

[8] Morley C. A., Perrins E. J., Grant P., Chan S. L., McBrien D. J., Sutton R. (1982). Carotid sinus syncope treated by pacing. analysis of persistent symptoms and role of atrioventricular sequential pacing. *Heart*.

[9] Madigan N. P., Flaker G. C., Curtis J. J., Reid J., Mueller K. J., Murphy T. J. (1984). Carotid sinus hypersensitivity: beneficial effects of dual-chamber pacing. *The American Journal of Cardiology*.

[10] Stang M. T., Armstrong M. J., Ogilvie J. B. (2012). Positional dyspnea and tracheal compression as indications for goiter resection. *Archives of Surgery*.

[11] Simantirakis E. N., Schiza S. I., Marketou M. E. (2004). Severe bradyarrhythmias in patients with sleep apnoea: the effect of continuous positive airway pressure treatment: a long-term evaluation using an insertable loop recorder. *European Heart Journal*.

[12] Grimm W., Hoffmann J., Menz V. (1996). Electrophysiologic evaluation of sinus node function and atrioventricular conduction in patients with prolonged ventricular asystole during obstructive sleep apnea. *American Journal of Cardiology*.

[13] Müller P. E., Kabus S., Robens E., Spelsberg F. (2001). Indications, risks, and acceptance of total thyroidectomy for multinodular benign goiter. *Surgery Today*.

